# Limitations of human t-lymphotropic virus type 1 antibody testing in hospitals of endemic regions in China

**DOI:** 10.3389/fcimb.2025.1474526

**Published:** 2025-04-03

**Authors:** Yi Chen, Yanxin Chen, Jing Zheng, Jiajie Yang, Yong Wu, Jianda Hu, Zhengjun Wu

**Affiliations:** ^1^ Fujian Provincial Key Laboratory on Hematology, Fujian Medical University Union Hospital, Fujian Institute of Hematology, Fuzhou, Fujian, China; ^2^ Department of Hematology, The Second Affiliated Hospital of Fujian Medical University, Quanzhou, Fujian, China; ^3^ Institute of Precision Medicine, Fujian Medical University, Fuzhou, Fujian, China

**Keywords:** human T-lymphotropic virus type 1, antibodies, nucleic acid testing, hematology, epidemiology

## Abstract

**Purpose:**

This study aims to evaluate the sensitivity and specificity of human T-lymphotropic virus type 1 (HTLV-1) antibody testing within hospitals located in HTLV-1 endemic areas of China.

**Method:**

We performed a retrospective analysis of the clinical records and laboratory results for 1,147 patients who underwent HTLV-1 antibody testing using the Wantai HTLV-1 antibody detection kit and Polymerase Chain Reaction (PCR) testing for HTLV-1 nucleic acids, at Fujian Medical University Union Hospital between 2017 and 2023.

**Result:**

The study population comprised 674 males (58.8%) and 473 females (41.2%), with an age distribution ranging from 7 to 86 years, and a median age of 50 years. Of the patients, 81 (7.1%) tested positive for HTLV-1 antibodies, including 39 males and 42 females. Predominantly, these positive cases were identified within the hematology department (93.8%). The cases originated from several high-prevalence coastal regions in Fujian province, such as Pingtan Island, Fuqing, Changle, Lianjiang, Fuan, Shouning, Xiapu, Zhouning, Fuding, Jiaocheng, Xiuyu, and Licheng. According to current standards for interpreting positive results, only 79.6% of patients with adult T-cell leukemia/lymphoma (ATLL) confirmed by HTLV-1 nucleic acid testing presented positive antibody results. Comparison of HTLV-1 antibody and nucleic acid test results revealed that the antibody test possessed a sensitivity of 63.0% and a specificity of 94.8%. A receiver operating characteristic (ROC) curve analysis determined that a threshold of 0.335 signal-to-cutoff (S/CO) was optimal for classifying positive antibody test results, yielding a sensitivity of 86.3% and a specificity of 94.4%.

**Conclusion:**

The Wantai HTLV-1 antibody test kit, when utilized in hospitals within endemic regions, exhibits a high level of specificity. However, its sensitivity is found to be lacking when evaluated against the current standards for the interpretation of positive results. For patients with a high clinical suspicion of HTLV-1 infection-related diseases, it is crucial to conduct testing of HTLV-1 antibodies and nucleic acids.

## Introduction

Human T-cell lymphotropic virus (HTLV) is a cell-associated retrovirus, and four types have been identified to date. HTLV type 1 (HTLV-1) infection is a significant pathogenic factor in the development of adult T-cell lymphoma/leukemia (ATLL) and is also a etiologic agent for various infectious disorders, including HTLV-associated myelopathy/tropical spastic paraparesis (HAM/TSP), HTLV-associated uveitis, and infective dermatitis (Proietti et al., 2005; Gessain and Cassar, 2012; Chan et al., 2017; Nozuma and Jacobson, 2019). According to the related literature, HTLV-1 exhibits several endemic zones across the globe. In Japan, the infection prevalence rate of HTLV-1 is approximately 0.07% to 4.6%. In the Caribbean islands, it is around 0.2%. In South America, the rate is about 0.1%, and it is estimated to be between 0.1% and 2.1% in certain regions of the Middle East (Hindawi et al., 2018; Iwanaga, 2020; Gessain et al., 2023; Valcarcel et al., 2023). In China, the prevalence of HTLV-1 infection is generally considered to be low, with reported cases being isolated, particularly in coastal areas of Fujian Province, where the prevalence rate is estimated to be 0.036% (Du et al., 2014; Chen et al., 2019; Zhao et al., 2020; Chang et al., 2021).

The current methods for detecting HTLV-1 involve serological assays such as enzyme-linked immunosorbent assay (ELISA) or chemiluminescence assay (CMIA) for initial screening, followed by confirmatory tests like Western blot or polymerase chain reaction (PCR) assays for the detection of viral nucleic acid (Itabashi et al., 2020; Wolf et al., 2022; Ji et al., 2023). The results of HTLV-1 antibody testing primarily arise from the screening of healthy blood donors or pregnant women and are known to be highly sensitive and specific (San Martín et al., 2016; Hindawi et al., 2018; Itabashi et al., 2020). Typically, for cases with negative initial antibody testing, Western blot or PCR is not performed as a confirmatory test. The Wantai HTLV-1 antibody test kit is extensively employed within the blood donor population in China, having become the predominant screening tool in hospitals for the exclusion of HTLV-1 associated diseases. However, the efficacy of this HTLV-1 antibody testing in hospital populations needs to be further clarified, and there has been a lack of comprehensive data analyzing the sensitivity and specificity of this HTLV-1 antibody testing in high-prevalence regions of China.

Therefore, the primary aim of this study is to assess the efficacy of the current antibody testing methods for HTLV-1 in hospitals situated within endemic areas of China.

## Methods

### Patients

Fujian Province is located in the southeastern coast of China, with a total area of about 121,400 square kilometers and a permanent resident population of 41.88 million. This study was conducted at Fujian Medical University Union Hospital, which had the largest hematology center in Fujian Province. The study included patients who were suspected of having HTLV-1 infection and had undergone testing for HTLV-1/2 antibodies, as well as individuals who had received clinical and laboratory follow-up and were identified as HTLV-1 carriers, from January 2016 to June 2023. Their medical records were reviewed retrospectively for clinical data and laboratory test outcomes. The study was approved by the Ethics Committee of Fujian Medical University Union Hospital (2024KY128) and was conducted in accordance with the ethical guidelines outlined in the 1964 Declaration of Helsinki.

### Assays for HTLV-1/2 antibody screening

All HTLV1/2 antibody detection tests were performed in our laboratory department by using an enzyme-linked immunosorbent assay (ELISA: Beijing Wantai Biological Pharmacy Enterprise Co., Beijing, China). Briefly, 212 amino acids from the envelope region (aa 185–396) of HTLV type 1 fused to 26 amino acids from the envelope region (aa 185–210) of HTLV type 2 were expressed in E. coli. The purified recombinant polypeptide was labeled with horse radish peroxidase (HRP) and coated onto microtiter plate. The assays were performed according to the manufacturers’ instruction, which indicated that serum samples that yielded an OD/cut-off ≥1.0 S/CO were considered positive for HTLV-1/2 antibodies. The sensitivity and specificity of the test as described by the manufacturer were 100% and 99.97%, respectively, when applied to the blood donation population.

### Confirmatory test

The confirmatory test for HTLV-1 infection was carried out in the hematology laboratory using PCR method. A human T lymphoblastic leukemia cell line, Jurkat, which was uninfected with HTLV-1, served as the negative control, while a patient with ATLL, confirmed by Sanger sequencing, acted as the positive control. Peripheral blood mononuclear cells (PBMCs) were isolated from patients’ whole blood using Ficoll-Paque density gradient centrifugation. Genomic DNA was extracted from the PBMCs and Jurkat cell line using a column extraction kit (Tiangen, Beijing, China) or the phenol-chloroform DNA isolation method. The concentration of the purified DNA was determined using a NanoDrop 1000 spectrophotometer (Thermo Scientific, Massachusetts, USA). A dilution of 400 ng of DNA from each sample was subjected to an “in-house” PCR for HTLV-1, with the amplification regions as previously described (Wu et al., 2016). In cases where there is a discrepancy between the detection of HTLV-1 antibodies and the nucleic acid analysis by agarose gel electrophoresis, we will proceed with further confirmation of the diagnosis using TaqMan real-time fluorescent quantitative PCR assay. The detection thresholds for nucleic acid analysis by agarose gel electrophoresis and real-time fluorescent quantitative PCR were 5 × 10^-2 ng/μL and 5 × 10^-4 ng/μL, respectively. All aspects of primer design, PCR experimental procedures, and criteria for result interpretation were detailed in **Supplementary File 1**. Patients with positive results from HTLV-1 detection by the PCR method were classified as true positive cases, whereas those with negative results were deemed true negative cases.

### Statistical analysis

Differences between patient subgroups were assessed using the chi-square test. HTLV-1 antibody and nucleic acid test results were visualized and analyzed using GraphPad Prism 9.5 software (GraphPad Software, San Diego, CA, USA). A Receiver Operating Characteristic (ROC) curve was constructed using R software (version 4.32; R Project for Statistical Computing, Vienna, Austria). In the ROC curve, the HTLV-1 nucleic acid testing results were used as the gold standard. Statistical significance between two groups was determined at P < 0.05.

## Results

### Patient characteristics

This retrospective study encompassed 1,147 patients, with 674 males (58.8%) and 473 females (41.3%). The patients’ ages ranged from 7 to 86 years, with a median age of 50 years. The majority population consisted of individuals aged 40 and above, accounting for 70.2% of the total cases. The inpatient department accounted for 84.0% of the total patients, and 16.0% were from the outpatient department. The hematology department was the primary source of cases, accounting for 92.2% of all cases. A small percentage of cases originated from the neurology (4.7%), infectious diseases (0.7%), emergency medicine (0.4%), and dermatology departments (0.1%). Details of patients’ characteristics were summarized **Table 1**.

**Table 1:** Patients characteristics and test results of HTLV-1 antibody.

Parameters	Number, (%)	Positive, n (%)	P-value
Total	1147	81 (7.1)	/
Sex			
Male	673 (58.7)	39 (5.8)	0.0475
Female	474 (41.3)	42 (8.9)	
Age, year	50 (7 - 89)		
<14	13 (1.1)	0 (0)	0.0454
≥14, <30	179 (15.6)	6 (3.4)	
≥30, <40	150 (13.1)	14 (9.3)	
≥40, <60	466 (40.6)	42 (9.0)	
≥60	339 (29.6)	19 (5.6)	
Patient sources			
Inpatient	963 (84.0)	40 (4.2)	<0.0001
Outpatient	184 (16.0)	41 (22.3)	
Department			
Hematology	1058 (92.2)	76 (7.2)	0.1361
Neurology	54 (4.7)	0 (0)	
Emergency medicine	8 (0.7)	2 (25.0)	
Infectious diseases	5 (0.4)	1 (20.0)	
Oncology	1 (0.1)	0 (0)	
Dermatology	1 (0.1)	0 (0)	
Others	20 (1.7)	2 (10.0)	

HTLV-1, human T-lymphotropic virus type 1.

### Antibody testing results

According to current standards for interpreting positive results as described by the manufacturer. A total of 81 patients (7.1%) tested positive for HTLV-1 antibodies, consisting of 39 males and 42 females. The majority of these positive cases (93.8%) were from the hematology department, and the details were summarized **Table 2**. The highest positive detection rate was observed in patients aged 40 to 60 years, at 9.4%. Females exhibited a higher positive detection rate (9.2%) compared to males (5.8%) (P = 0.0398). The primary reasons for conducting antibody testing included rash, fever, and lymphadenopathy, as well as elevated serum calcium and lymphocyte counts. Patients with accompanying rash had the highest positive rate at 26.4%. Notably, only 79.6% of patients with ATLL confirmed by HTLV-1 PCR testing presented positive antibody results. Additionally, 25.4% of the immediate family members of ATLL patients displayed positive antibody results, including patients’ parents, siblings and children. Two cases with HAM/TSP confirmed by HTLV-1 PCR testing presented negative antibody results.

**Table 2:** Characteristics of positive samples in hematology department.

Parameters	Number, (%)	Positive rate (%)	P-value
Total	1058 (100.0)	76 (7.2)	/
Age, year			
<40	309 (29.2)	18 (5.8)	0.0744
≥40, <60	413 (39.0)	39 (9.4)	
≥60	336 (31.8)	19 (5.7)	
Sex			
Male	622 (58.8)	36 (5.8)	0.0398
Female	436 (41.2)	40 (9.2)	
Symptoms and laboratory examinations, n (%)			
Skin rash	53 (5.0)	14 (26.4)	<0.0001
Enlarged lymph nodes	513 (48.5)	40 (7.8)	
Fever	233 (22.0)	8 (3.4)	
Elevated serum calcium	255 (24.1)	45 (17.6)	
Increased lymphocyte count	129 (12.2)	25 (19.4)	
Diagnosis			
ATLL	58 (5.5)	45 (77.6)	<0.0001
Immediate family members of ATLL	114 (10.8)	29 (25.4)	
Others	890 (84.1)	4 (0.4)	

ATLL, adult T cell leukemia/lymphoma.

### Geographical distribution of patients

**Figure 1:**
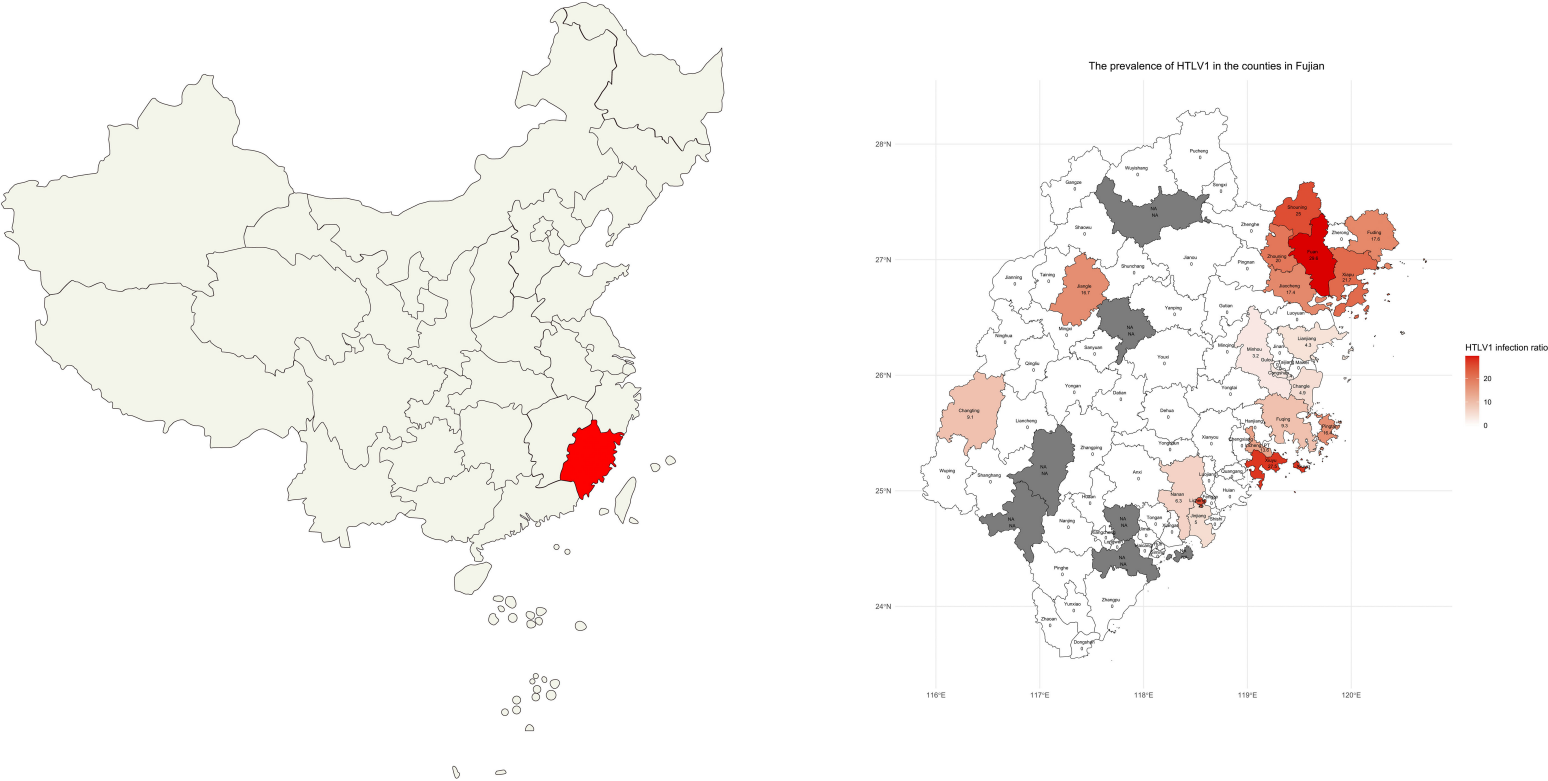
Distribution of Patients’ Living Areas. A map depicting the geographical distribution of the patients’ living areas in Fujian province, highlighting the coastal regions of Pingtan Island, Fuqing, Changle, Lianjiang, Fuan, Shouning, Xiapu, Zhouning, Fuding, Jiaocheng, Xiuyu, and Licheng with a higher prevalence of HTLV-1. NA: no data was available because no patient from this region was tested for HTLV-1.

The majority of patients with positive antibody results hailed from several high-prevalence coastal regions in Fujian province (Du et al., 2014; Chen et al., 2019; Zhao et al., 2020; Chang et al., 2021), as they were summarized at **Figure 1** and **Table 3**. In Fuzhou City, Pingtan Island had the highest positive rate at 16.4%, followed by Fuqing, Changle, Lianjiang, and Minhou, with rates of 9.3%, 4.9%, 4.3%, and 3.2%, respectively. In Ningde city, the respective positive rates were as follows: Fuan (29.6%), Shouning (25.0%), Xiapu (21.7%), Zhouning (20.0%), Fuding (17.6%), and Jiaocheng (17.4%). Xiuyu and Licheng in Putian City were also high-prevalence areas, with rates of 27.5% and 13.6%, respectively. No positive cases were detected in the coastal city of Xiamen, and the positive rates in other inland regions were relatively low in this study.

**Table 3:** Distribution of patients' living areas.

Regions	Population (Million)	Number	Positive, n (%)
Fujian Province	41.88	1111	77 (6.9)
Fuzhou	8.29	426	23 (5.4)
Pingtan	0.4	67	11 (16.4)
Fuqing	1.39	75	7 (9.3)
Changle	0.79	41	2 (4.9)
Lianjiang	0.64	23	1 (4.3)
Minhou	0.99	31	1 (3.2)
Cangshan	1.14	29	1 (3.4)
Ningde	3.15	151	28 (18.5)
Fuan	0.61	27	8 (29.6)
Shouning	0.18	8	2 (25.0)
Xiapu	0.48	46	10 (21.7)
Zhouning	0.15	5	1 (20.0)
Fuding	0.55	17	3 (17.6)
Jiaocheng	0.62	23	4 (17.4)
Others	0.70	16	0 (0.0)
Putian	3.21	147	22 (15.0)
Xiuyu	0.32	69	19 (27.5)
Licheng	0.49	22	3 (13.6)
Others	2.40	56	0 (0.0)
Quanzhou	8.78	122	3 (2.5)
Sanming	2.49	58	1 (1.7)
Nanping	2.68	91	0 (0.0)
Longyan	2.72	57	0 (0.0)
Zhangzhou	5.05	43	0 (0.0)
Xiamen	5.16	16	0 (0.0)
Other provinces*	NA	36	4 (11.1)

*In this study, a total of 36 patients from outside Fujian Province underwent HLTV-1 antibody testing. These patients hailed from 14 other provinces and regions. Due to the small number of tests conducted in each area, the total population of these regions was not displayed. NA, not available.

### Comparison of antibody and nucleic acid testing for HTLV-1

A total of 216 patients underwent both HTLV-1 antibody and nucleic acid testing, and the results were showed at **Table 4**. Of these, 81 patients with positive HTLV-1 nucleic acid results (true positive cases) were identified. It was observed that only 51 of these cases (63.0%) were positive in the antibody tests, while 30 cases (37.0%) were negative. The remaining 135 patients with negative HTLV-1 nucleic acid results were considered true negative cases. Among these, 7 cases (5.2%) were positive and 128 cases (94.8%) were negative in antibody testing. In summary, the true positive and false positive rates were 87.9% and 12.1%, respectively, with a sensitivity of 63.0% and a specificity of 94.8%.

**Table 4:** Results of HTLV-1 antibody and nucleic acid testing in 216 cases.

Methods	Results	
	Nucleic acid testing	
Antibody testing	Positive	Negative
Positive	51	7
Negative	30	128

HTLV-1, human T-lymphotropic virus type 1. The true positive rate was 87.9%, and the false positive rate was 12.1%. The sensitivity was 63.0%, and the specificity was 94.8%.

### Adjustment of positive criteria for antibody detection

**Figure 2:**
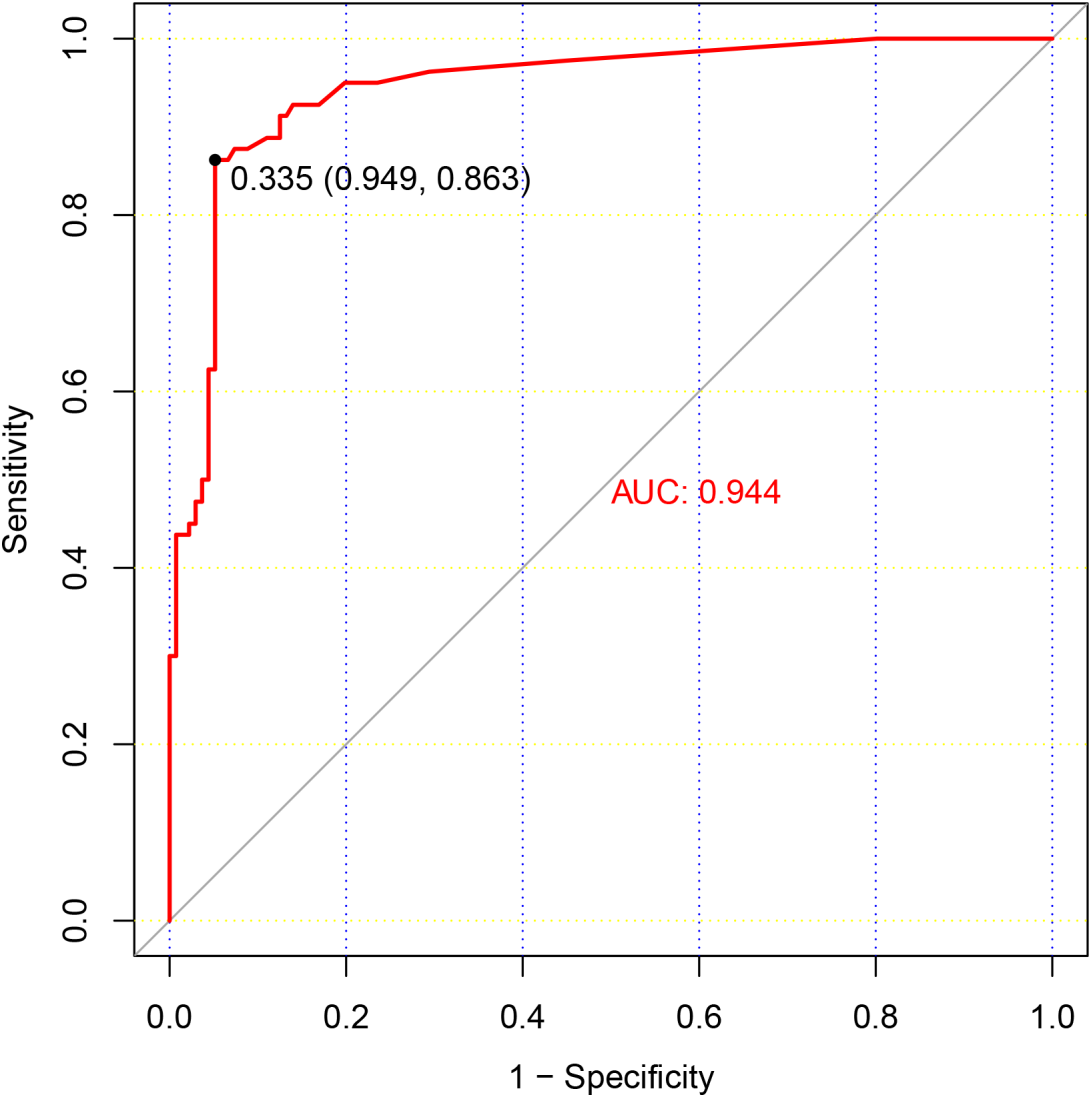
ROC Curve Analysis of HTLV-1 Antibody Testing. This figure presents a ROC curve analysis for HTLV-1 antibody testing, depicting the relationship between the sensitivity and specificity of various threshold values. The results of HTLV-1 nucleic acid testing served as the reference standard, with patients testing positive for HTLV-1 nucleic acid being classified as true positive cases. The optimal cut-off value, which maximizes the area under the ROC curve, is indicated by a dashed line at a S/CO ratio of 0.335.

**Figure 3:**
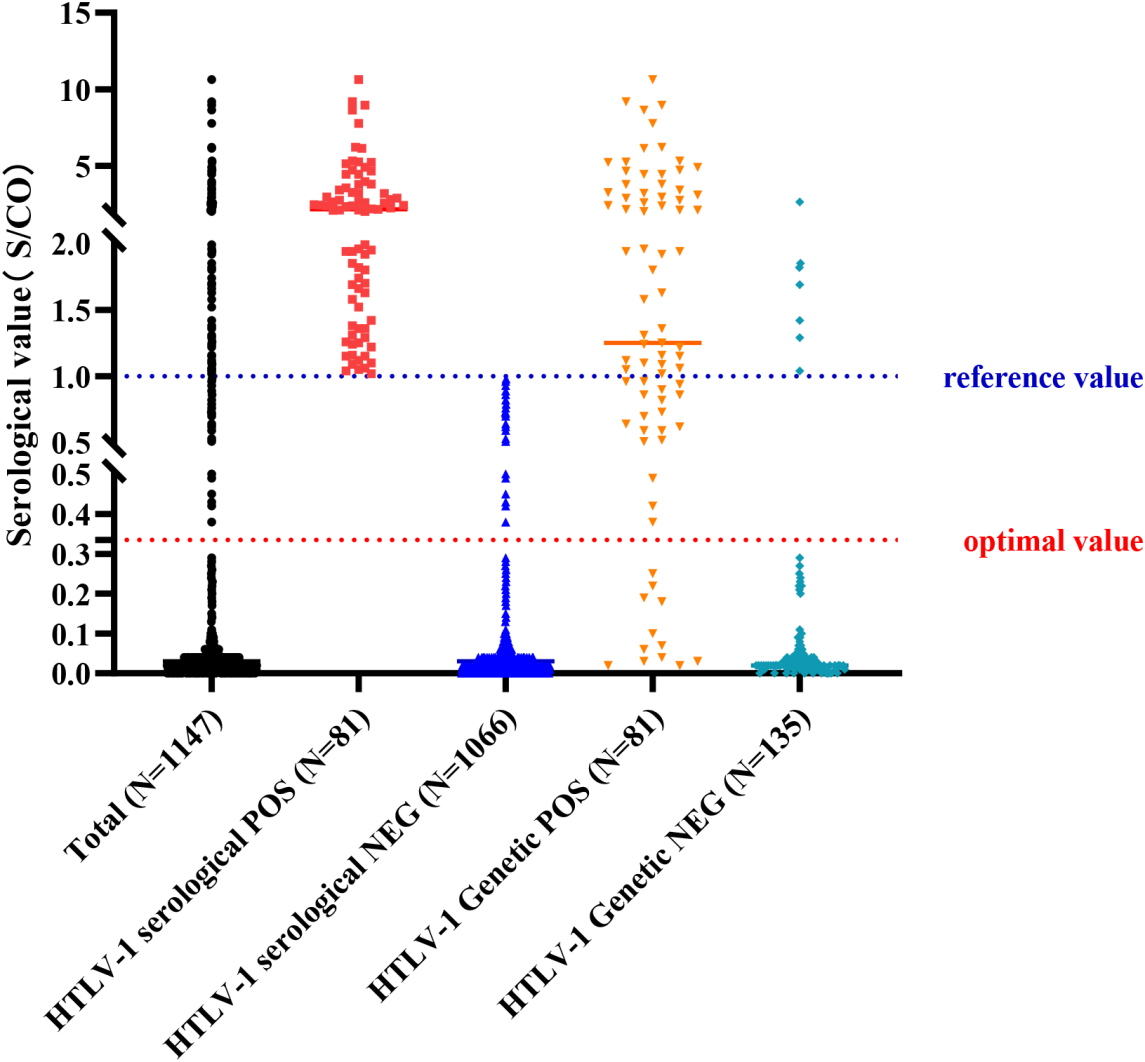
Results of Antibody and Nucleic Acid Testing of HTLV-1. A scatter plot summarizing the results of HTLV-1 antibody and nucleic acid testing in the study population, showing the positive and negative rates for each testing method. Reference value: positive judgment criteria described by HLTV-1 test kit; optimal value: positive judgment criteria adjusted with ROC curve.

**Figure 4:**
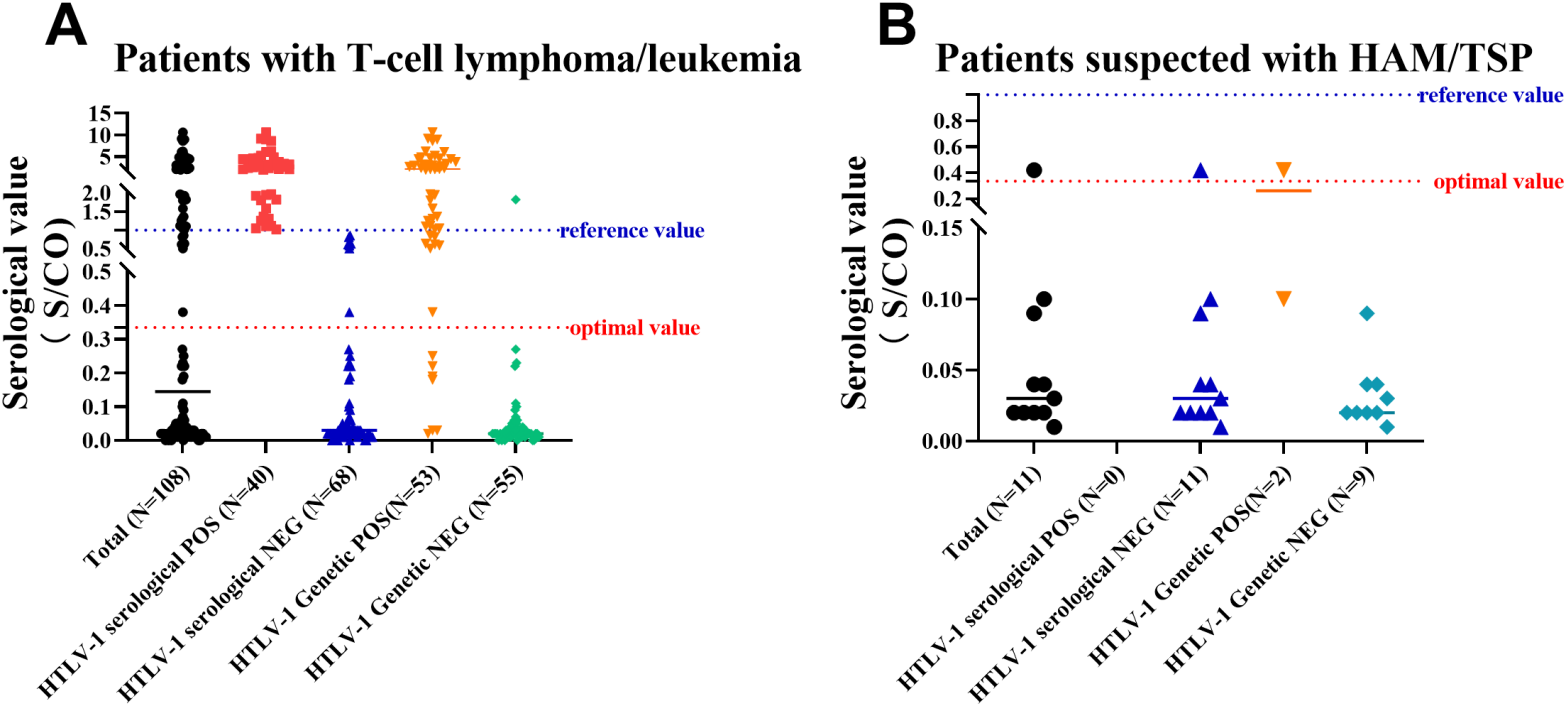
Results of Antibody and Nucleic Acid Testing of HTLV-1 in Patients with T-Cell Lymphoma/Leukemia or Suspected with HAM/TSP. **(A)** A scatter plot of results for patients with T-cell lymphoma/leukemia, showing the distribution of positive and negative antibody and nucleic acid test results. **(B)** A scatter plot of results for patients suspected with HAM/TSP, illustrating the distribution of positive and negative antibody and nucleic acid test results. Reference value: positive judgment criteria described by HLTV-1 test kit; optimal value: positive judgment criteria adjusted with ROC curve.

Observing the high specificity (94.8%) but lower sensitivity (63.0%) of antibody testing, it was deemed necessary to reevaluate the positive threshold. A Receiver Operating Characteristic (ROC) curve analysis was performed to determine the optimal cut-off value for positive antibody detection (**Figure 2**). The analysis revealed that setting the cut-off value at 0.335 S/CO maximized detection efficiency, yielding a sensitivity of 86.3% and a specificity of 94.4%. However, for results below this threshold, there remained a 13.7% chance of a false negative result. HTLV-1 antibody testing results for the overall patient cohort and subgroups, including those with T-cell Lymphoma/Leukemia and those suspected with HAM/TSP based on current and revised positive criteria were summarized at **Figures 3**, **4**.

### Dynamic monitoring of HTLV-1 antibody and nucleic acid levels

**Figure 5:**
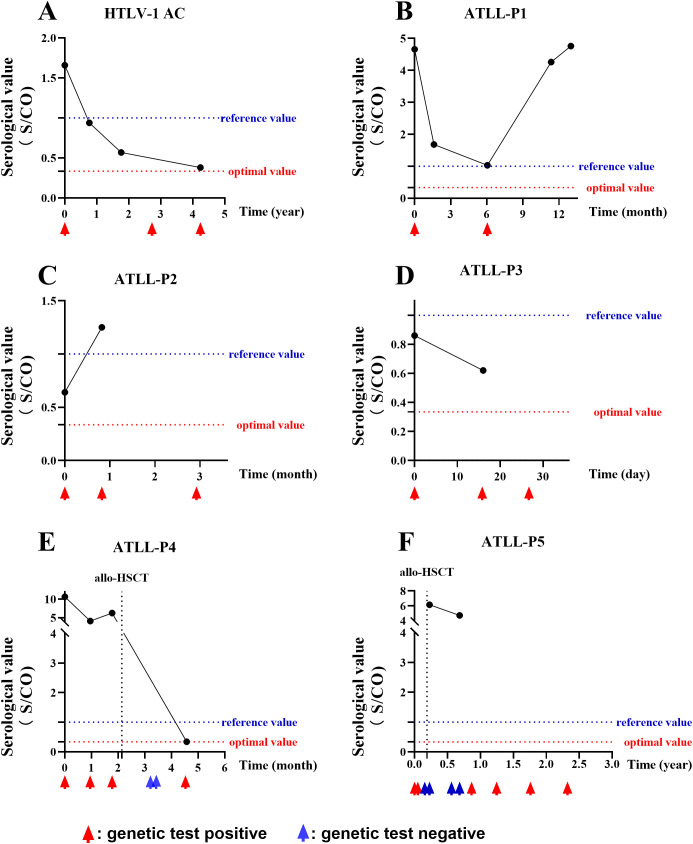
Follow-up of HTLV-1 Antibody and Nucleic Acid. A series of graphs depicting the dynamic changes in HTLV-1 antibody and nucleic acid levels over time in various patient cohorts. **(A)** A HTLV-1 carrier showing persistent positive results. **(B-D)** Three patients with ATLL, each showing persistent positive antibody and nucleic acid results. **(E, F)** Two patients with ATLL who received allo-HSCT, with transient negative nucleic acid results post-transplant but persistent positive antibody results. Reference value: positive judgment criteria described by HLTV-1 test kit; optimal value: positive judgment criteria adjusted with ROC curve.

In the present study, a subset of patients underwent multiple HTLV-1 antibody and nucleic acid tests throughout their disease course. We observed that the antibody levels could fluctuate in certain cases, and the interpretation of these results varied significantly based on the current positive criteria. By adjusting the cut-off value for positive criteria to 0.335 S/CO, we noted that HTLV-1 carriers and patients with ATLL consistently presented positive results for both antibody and nucleic acid tests, even in the absence of allogeneic hematopoietic stem cell transplantation (allo-HSCT), as illustrated in **Figures 5A-D**. In contrast, for patients with ATLL who underwent allo-HSCT, HTLV-1 nucleic acid levels were undetectable within a specific timeframe post-transplant, yet antibody testing remained persistently positive, as depicted in **Figures 5E, F**.

## Discussion

HTLV-1 is rare in most regions of China, but it is locally prevalent in coastal areas of Fujian province (Chang et al., 2021). Our team was among the first to report ATLL cases as early as 1992 (Chen et al., 1992), and established a genetic diagnostic system for HTLV-1 and ATLL in 1996 (Hu et al., 1996). To our knowledge, we are currently the only hospital in Fujian province that conducts HTLV-1 viral nucleic acid testing. We have published the largest sample size of ATLL case reports in China (Luo et al., 2024), and HAM/TSP is also reported in our center recently. Currently, antibody testing for HTLV-1 plays a crucial role in identifying individuals who may be afflicted with related diseases. The Wantai HTLV-1 antibody test kit has emerged as the leading screening method utilized in clinical practice for this purpose. However, limited data are available regarding the efficacy of this antibody testing in hospitals situated within HTLV-1 endemic areas of China, and our study contributes to the existing literature by reporting on a substantial number of cases within this setting.

In developed and some developing countries where HTLV-1 is endemic, laboratory screening for HTLV-1/2 infection has become standard practice for blood donors. Antibody testing of HTLV-1/2 is the most commonly employed method (Canavaggio et al., 1990; Fujino et al., 1991; Laperche et al., 2017). Wantai HTLV ELISA kit was one of the main kits in several large-scale epidemiological studies of HTLV-1 infection in China (Du et al., 2014; Chan et al., 2017; Zhao et al., 2020; Chang et al., 2021), and its sensitivity and specificity are 100% and 99.97% in blood donation population. Currently, this HTLV ELISA Kit has been widely adopted for detecting HTLV-1 infection in China. However, we must know that the blood donation population consists of young and healthy individuals, whereas the hospital population includes elderly patients, and patients with immune suppression or immune deficiency due to the disease itself or medical treatment.

Our study observed that the majority of cases originated from the hematology department, primarily among patients suspected or confirmed to have ATLL, or among immediate family members of ATLL patients. Among these high-risk groups for HTLV-1 infection, we identified 81 positive cases through antibody testing. Notably, only 45 out of 58 patients confirmed with ATLL and none out of 2 patients confirmed with HAM/TSP exhibited positive antibody test results for HTLV-1. This suggests that low detection sensitivity by the current ELISA test for HTLV-1 in hospital populations. Therefore, in clinical settings where there is strong suspicion of HTLV-1-related diseases based on symptoms and other laboratory results, it is crucial to conduct confirmatory nucleic acid testing for HTLV-1 through PCR. Moreover, approximately one-fourth of the immediate family members of ATLL patients in our data exhibited positive results for HTLV-1 antibody testing, warranting long-term follow-up for this group.

Comparing antibody testing to PCR testing of HTLV-1, we observed that antibody testing exhibited high specificity but low sensitivity. This implies that individuals with negative antibody testing results are at low risk of HTLV-1 infection, but a considerable proportion of HTLV-1-infected individuals may have negative antibody test results. As we mentioned above, there were significant differences in biological characteristics between the blood donation population and the hospitalized population, so the positive criteria currently based on blood donation population might not be appropriate for the hospital population. Thus, to determine the better optimal positive cut-off value for antibody detection, we conducted a ROC curve analysis. The analysis revealed that adjusting the positive cut-off value to 0.335 S/CO maximized detection efficacy. Additionally, techniques such as CMIA, WB, and Line Immunoassay (LIA) provide valuable confirmatory evidence for HTLV-1 infection, and their use should be considered when appropriate.

Previous studies have indicated that HTLV-1 cause a life-long infection and cannot be cleared (Vallinoto et al., 2019; Zuo et al., 2023). Our findings suggested that among HTLV-1 carriers and patients with ATLL, both antibody and nucleic acid testing remained persistently positive. Interestingly, in some patients with ATLL who underwent allogeneic hematopoietic stem cell transplantation (allo-HSCT), HTLV-1 nucleic acid became undetectable shortly after transplantation, while the antibody levels showed a decreasing trend but persistent positive. This could be attributed to the transplantation process potentially eliminating a majority of the virus, resulting in extremely low viral loads (Okamura et al., 2007). Thus, the interplay between different diagnostic strategies is an important consideration in the comprehensive management of HTLV-1 infection.

Certain limitations should be considered when interpreting the study results. Firstly, this investigation was a single-center retrospective analysis primarily focused on patients within the hematology department, which may not reflect the broader patient population across various medical settings. Secondly, the living areas of the patients and their corresponding positive rates of HLTV-1 testing can indicate the epidemic areas of HLTV-1 infection, but it cannot represent the infection rate in these specific regions. Thirdly, our study was limited by the availability of testing reagents, which necessitated the use of only one of the currently prevalent antibody detection methods. Moreover, the sample size used to refine the positive criteria for antibody testing was relatively small, highlighting the need for additional research to determine the most appropriate threshold for a positive antibody test result.

In conclusion, our study emphasizes that the use of the Wantai test kit for HTLV-1 antibody screening in hospitals situated within HTLV-1 endemic areas exhibits a high degree of specificity. However, it is noted that this kit’s sensitivity is insufficient when evaluated against the current standards for the interpretation of positive results. Revising the positive cutoff threshold for HTLV-1 antibody testing to 0.335 S/CO could improve the sensitivity of the assay. In clinical settings where HTLV-1 infection-associated diseases are strongly suspected, it is imperative to concurrently assess for both HTLV-1 antibodies and nucleic acids.

## Data Availability

The original contributions presented in the study are included in the article/**Supplementary Material**. Further inquiries can be directed to the corresponding authors.
