# Supplementary material for: Limitations of human t-lymphotropic virus type 1 antibody testing in hospitals of endemic regions in China
Source: Front Cell Infect Microbiol. 2025 Apr 3;15:1474526. doi: 10.3389/fcimb.2025.1474526 (PMC12003307; doi:10.3389/fcimb.2025.1474526)
Supplement: Supplementary file 1 [file Table1.docx]

**Agarose gel electrophoresis of nucleic acid**

In the experimental setup, β-globin was employed as an internal control to ensure the quality of the nucleic acid samples. The PCR strategy employed two sets of primers, tax and pol, for the detection of HTLV. These primers were designed to differentiate between HTLV-1 and HTLV-2. The tax primer was capable of detecting both HTLV-1 and HTLV-2, while the pol primer was specific to HTLV-1. Primers used in the PCR assay are summarized at Supplementary Table 1.

| Supplementary Table 1. Primers used in the PCR assay. | | |
| --- | --- | --- |
| Primer Name | sequence (5’→3’) | Products |
| β-globin+ | CAACTTCATCCACGTTCACC | 268bp |
| β-globin- | GAAGAGCCAAGGACAGGTAC |  |
| pol+ | CTTCACAGTCTCTACTGTGC | 119bp |
| pol- | CGGCAGTTCTGTGACAGGG |  |
| tax+ | CGGATACCCAGTCTACGTGT | 159bp |
| tax- | GAGCCGATAACGCGTCCATCG |  |

The PCR reactions were carried out using a Thermal Cycler 2720 (Applied Biosystems) with the following components: 12.5 µL of 2× PCR Master Mix (Thermo Scientific), 0.4 µM of final concentration for each primer, 400 ng of template DNA, and distilled water added to reach a total volume of 25 µL. The PCR process involved three steps and consisted of:

1. An initial denaturation step at 95℃ for 15 minutes to activate the enzymes.

2. Thirty-five amplification cycles, each consisting of:

- 30 seconds at 95℃ for denaturation,

- 30 seconds at 60℃ for annealing,

- 30 seconds at 72℃ for extension.

3. A final extension step at 72℃ for 7 minutes to ensure complete amplification of the target sequences.

The amplifications of each target processed in single reactions. The PCR products were analyzed by a 3% agarose gel electrophoresis and visualized using the ChemiDoc Imaging System ([Bio-Rad](https://www.bio-rad.com/corporate/about-bio-rad?ID=1003)) after the ethidium bromide staining. During nucleic acid gel electrophoresis, the results were interpreted as follows: if both the tax and pol were positive when compared to the positive control, the HTLV-1 nucleic acid test result was classified as positive. Conversely, if both the tax and pol were negative when compared to the negative control, the HTLV-1 nucleic acid test result was classified as negative.

**Quantification of HTLV-1 Proviral Load (PVL) Using TaqMan Real-Time Fluorescent Quantitative PCR**

To ascertain the proviral load (PVL) of cases with discordant results from serological and nucleic acid amplification tests, we utilized a more sensitive molecular approach: the TaqMan real-time fluorescent quantitative PCR assay. To establish standards for quantifying HTLV-1 copies and determining cell equivalents, we employed serially diluted (10-fold) genomic DNA from TL-om1 cells, a well-characterized HTLV-1-positive cell line harboring a single copy of HTLV-1 and two copies of the RNase P gene per diploid genome [1]. These standards facilitated the accurate measurement of both viral copies and cell concentration.

Specific primers and a probe targeting the highly conserved pX region of HTLV-1 were used to quantify the viral copy number, while a distinct set of primers and probe targeting the RNase P gene served as an internal control for cell number quantification. In preparation for the assay, a 20 µL TaqMan real-time PCR reaction mixture was assembled, based on a previously described protocol [3] with slight modifications: the mixture contained 10 µL of 2 × TaqMan Universal Master Mix (Applied Biosystems), 8 µL of DNA template with the appropriate standards or 100 ng of the sample DNA to be assayed, a final concentration of 0.4 mM of each primer, and 0.2 mM of each probe.

All samples, standards, negative, and positive controls were run in triplicate to ensure reliability. The real-time PCR was executed on a Quant Studio 5 Real-Time PCR System (Applied Biosystems) using a two-step thermal cycling protocol: an initial enzyme activation step at 95℃ for 10 minutes, followed by 45 amplification cycles, each consisting of 15 seconds at 95℃ and 45 seconds at 60℃ with fluorescence detection.

The copy numbers of HTLV-1 pX and RNase P gene were determined based on their respective standard curves. Subsequently, the HTLV-1 PVL was normalized using the following formula:

PVL = (pX copy number / RNase P copy number) × 2 × 10^2

The PVL was expressed as the number of HTLV-1 copies per 10^2 peripheral blood mononuclear cells (PBMCs). The sequences of the primers and probes used are provided in Supplementary Table 2.

Supplementary Table 2: Primers and Probes Employed in the TaqMan Real-Time PCR Assay.

| Primers and probes | sequence (5’→3’) |
| --- | --- |
| hRPPH1-S | 5’-GGAGCTTGGAACAGACTCAC-3’ |
| hRPPH1-AS | 5’-GGAGAGTAGTCTGAATTGGGTTATG-3’ |
| hRPPH1-Probe | 5`6-FAM-CCTCACCTCAGCCATTGAACTCACTTC-3`BHQ1 |
| pX-S | 5’-CGGATACCCAGTCTACGTGTT-3’ |
| pX-AS | 5’-CAGTAGGGCGTGACGATGTA-3’ |
| pX-Probe | 5`6-FAM-CTGTGTACAAGGCGACTGGTGCC-3`BHQ1 |

**Reference**

1. Zhao T, Wang Z, Fang J, et al. HTLV-1 activates YAP via NF-κB/p65 to promote oncogenesis. Proc Natl Acad Sci U S A. 2022 Mar 1;119(9):e2115316119.
2. .Kuramitsu M, Okuma K, Yamagishi M, et al. Identification of TL-Om1, an adult T-cell leukemia (ATL) cell line, as reference material for quantitative PCR for human T-lymphotropic virus 1. J Clin Microbiol. 2015 Feb;53(2):587-96.

3.Naderi M, Paryan M, Azadmanesh K, et al. Design and development of a quantitative real time PCR assay for monitoring of HTLV-1 provirus in whole blood. J Clin Virol. 2012 Apr;53(4):302-7.
